# High-order harmonic generation from the dressed autoionizing states

**DOI:** 10.1038/ncomms16061

**Published:** 2017-07-17

**Authors:** M. A. Fareed, V. V. Strelkov, N. Thiré, S. Mondal, B. E. Schmidt, F. Légaré, T. Ozaki

**Affiliations:** 1 Institut national de la recherche scientifique—Centre Energie Matériaux Telécommunications, 1650 Lionel-Boulet, Varennes, Québec, Canada J3X 1S2; 2 A. M. Prokhorov General Physics Institute of RAS, Vavilova street 38, 119991 Moscow, Russia; 3 Moscow Institute of Physics and Technology (State University), 141700 Dolgoprudny, Moscow Region, Russia; 4 ELI-ALPS, ELI-Hu Kft., Dugonics ter 13, H-6720 Szeged, Hungary; 5 few-cycle, Inc., 2890 Rue de Beaurivage, Montreal, Quebec, Canada H1L 5W5

## Abstract

In high-order harmonic generation, resonant harmonics (RH) are sources of intense, coherent extreme-ultraviolet radiation. However, intensity enhancement of RH only occurs for a single harmonic order, making it challenging to generate short attosecond pulses. Moreover, the mechanism involved behind such RH was circumstantial, because of the lack of direct experimental proofs. Here, we demonstrate the exact quantum paths that electron follows for RH generation using tin, showing that it involves not only the autoionizing state, but also a harmonic generation from dressed-AIS that appears as two coherent satellite harmonics at frequencies ±2Ω from the RH (Ω represents laser frequency). Our observations of harmonic emission from dressed states open the possibilities of generating intense and broadband attosecond pulses, thus contributing to future applications in attosecond science, as well as the perspective of studying the femtosecond and attosecond dynamics of autoionizing states.

## Abstract (web summary)

High-energy photons in XUV range and attosecond pulses are generated from infrared laser pulses through high harmonic generation in gases and solids. Here, the authors demonstrate the microscopic origin of resonant harmonic generation involving the autoionizing states of Sn in plasma plumes.

High-order harmonic generation (HHG) is an excellent tabletop source of coherent extreme ultraviolet (XUV) and soft X-ray radiation. As high-order harmonics are intrinsically generated in attosecond bursts, it also opens a new domain of attosecond science^1^,^2^,^3^,^4^. More recently, high-harmonic spectroscopy is developing as a unique tool for studying the electronic structure of atoms and molecules^5^,^6^. The HHG process from most nonlinear media is well explained by the three-step model^7^. However, for some media, the intensity of a specific harmonic order could be enhanced by one to two orders of magnitude compared with the neighbouring harmonics, which are generated owing to conventional three-step process. This has been observed when the wavelength of a harmonic is in close proximity to an autoionizing resonance of the nonlinear medium^8^,^9^. Major efforts up to now have been devoted to increasing the photon flux of these resonant harmonics (RH)^8^,^10^,^11^. However, there have only been a few experiments that were aimed for better understanding of the physics involved behind this RH generation. These experiments have only shown that the RH intensity depends strongly on the ellipticity of the driving laser^11^, and follows phase matching conditions similar to other conventional gas harmonics^10^.

There have also been theoretical efforts aimed at better understanding the physics behind RH generation^12^,^13^,^14^,^15^,^16^,^17^. Different models have shown that the increase of the RH intensity can be attributed to the macroscopic processes^13^,^14^ (to improved phase matching of the RH generation) or to the microscopic ones^15^,^16^,^17^,^18^,^19^. In the latter direction, several different yet-to-be-verified theoretical models have been proposed using bound–bound^15^, bound-continuum transitions^16^ and the electronic motion in the continuum^17^ to explain the mechanism of RH generation. In particular, the four-step model, proposed by Strelkov^17^ has demonstrated an agreement with several experimental observations^20^,^21^. In this model, steps 1 and 2 are the same as for the three-step model. However, in step 3, the electron accelerated in the continuum is trapped by the parent ion, so that the system lands in the autoionizing state (AIS), and in step 4 relaxes to the ground state by emitting the harmonic photon. Experimentally observed intensity enhancements of RH have been compared with theory for several materials^17^, and the relative phase between RH and non-RH have been experimentally measured and compared with theory^18^. However, intensity and phase measurements of the harmonics are still indirect evidence of the four-step model, and thus there currently lacks direct and concrete evidence that the AIS is involved in RH generation.

In this work, we study the electron quantum paths in the vicinity of AIS with mid-IR tunable driving fields. Our results allow to unambiguously clarify the mechanism involved in RH generation. The present study shows that RH generation process involves the AIS for coherent harmonic emission at resonant energy via the microscopic response. Further, it has been revealed that the RH generation process involves the dressed-AIS for coherent harmonic emission at frequencies ±2Ω from the RH frequency (Ω represents laser frequency). As RH is an excellent candidate as a source for intense harmonics^8^,^9^, the involvement of dressed states in HHG opens the perspective to expand the bandwidth of RH (for example, we can generate harmonics at ±2Ω, ±4Ω, and above frequencies), thus opening the possibility to generate intense and ultrashort attosecond pulses, useful for the applications of attosecond science^1^,^2^. The direct involvement of AIS in RH generation via microscopic response will also provide opportunities to study emission dynamics of AIS with ultrafast lasers. For example, we can extract information about the absolute time that the electron stays in the AIS before emission, study electron–electron interaction, and interference of RH and direct harmonic at attosecond time scale by driving harmonics using pulses with certain duration, as the electron stays for a short time in the AIS. Further, harmonic emission from virtual quantum states will provide opportunities to understand the nature of virtual states and their influence on the physical systems both in physics and chemistry, hence providing better control on the systems and roots to find new applications.

## Results

### RH response with tunable driving wavelengths

**Figure 1:**
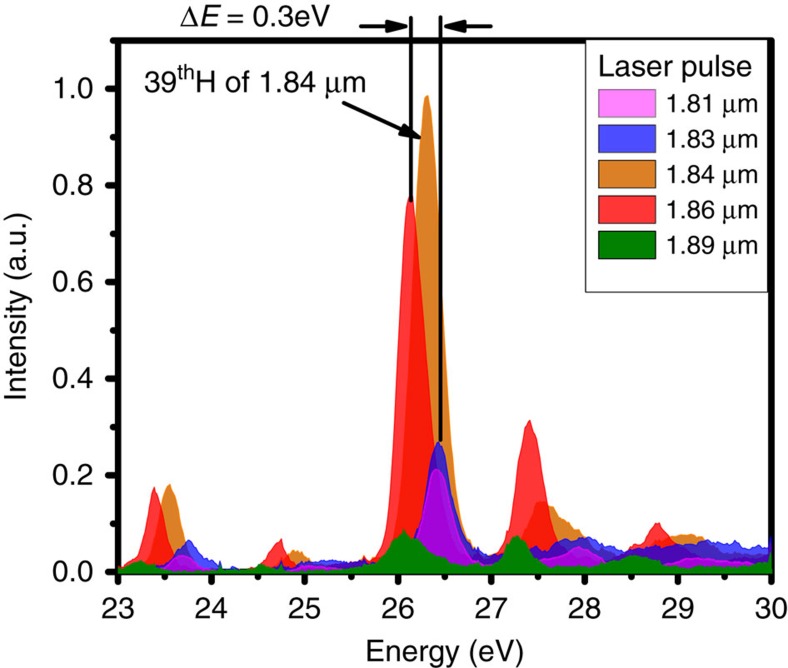
High-order harmonic spectra with tunable driving laser. The extreme-ultraviolet spectra measured at different driving laser wavelengths ranging from 1.81 μm to 1.89 μm. Maximum harmonic intensity is observed for a pulse centred at 1.84 μm, as this wavelength is exactly 39-photon resonant with the 4*d*^10^5*s*^2^5*p*→4*d*^9^5*s*^2^5*p*^2^ transition of Sn^+^. The harmonic intensity then decreases rapidly as the central driving laser wavelength is detuned. The driving laser intensity for these spectra is maintained at ∼1.3 × 10^14^ W cm^−2^.

We have observed variation in the intensity of the RH with the change in the central wavelength of the driving laser. As the driving laser used in this experiment is broadband (∼100 nm), we find that there is a certain wavelength range in which intensity enhancement of the RH could be observed^11^,^20^. We show in Fig. 1 the harmonic spectra from the Sn^+^ around the AIS resonance, when the central wavelength of the driving laser is varied from 1.81 to 1.89 μm. The intensity of the RH is maximum for driving laser at 1.84 μm central wavelength, as this wavelength is exactly 39-photon resonant with the 26.3 eV autoionizing resonance of Sn^+^. As we increase the detuning of the driving laser wavelength from this resonance, the RH intensity decreases. Only weak RH is observed at laser wavelengths of 1.81 and 1.89 μm, which disappears completely at 1.80 and 1.90 μm wavelengths. Therefore, these results show that when using driving lasers of ∼100 nm bandwidth, the RH of Sn^+^ responds well for at least ±30 nm of bandwidth, around the resonant wavelength of ∼1.84 μm.

### Microscopic response of RH

Our results allow defining if the observed enhancement of the RH intensity is due to the macroscopic or the microscopic processes. In Fig. 1, we observe that when harmonic is close to the resonance, the harmonic intensity is enhanced in the spectral region of ∼0.3 eV. The width of this spectral region is close to the width of the 4*d*^9^5*s*^2^5*p*^2^ (1D) ^2^D_5/2_ state of Sn^+^ (0.16 eV). The former is broader because of the non-zero harmonic line width and transition line broadening owing to AIS photoionization. The width of the enhancement region equal to 0.3 eV was found numerically^22^, although much shorter driving wavelength was used in these calculations. This shows that the enhancement in our conditions is due to the microscopic response enhancement and not due to the macroscopic effects because the region where the phase matching could be improved by the resonance is typically narrower than the resonance width, and the centre of this region is shifted with respect to the transition frequency. Moreover, we see that the harmonic below the resonance one is suppressed, in agreement with the microscopic response^22^. This effect was not previously observed in the HHG because the frequency range between the harmonics was larger due to higher driving frequency^20^.

### Splitting of the harmonic spectral line

**Figure 2:**
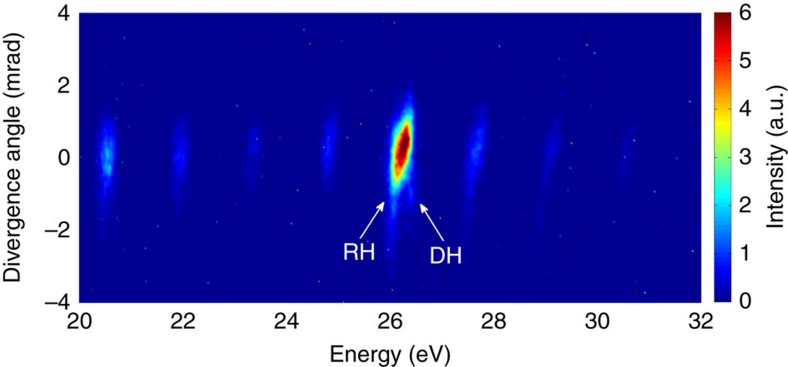
Overlap of resonant harmonic and direct harmonic at resonant energy. High-order harmonic spectrum of Sn^+^ at 1.75 μm driving laser wavelength, showing that the stronger resonant harmonic (RH) and relatively weak direct harmonic (DH) are overlapped with each other at energy∼26.3 eV. This spectrum is recorded at laser intensity of ∼1.43 × 10^14^ W cm^−2^.

More features observed under small detuning from the resonance can be seen in Fig. 2. It shows a typical high-order harmonic spectrum from Sn^+^ for driving lasers at 1.75 μm wavelength, which is in 37-photon resonance with the 26.3 eV autoionizing resonance^11^. We see that around energies of 26 eV there are actually two close peaks. The frequency of the more intense one is the transition frequency, whereas the frequency of the other is the 37th harmonic frequency. Such harmonic line splitting was theoretically predicted in ref. 18. This theory, however, assumes initial coherent superposition of states of the generating particle. Further theoretical study of the splitting in more realistic situation of initial population only in the ground state was done in ref. 22. This theory shows, in particular, that the line shape of the harmonic near the resonance is a product of the harmonic line that would be emitted in the absence of the resonance, and the factor that is unity far from the resonance and has a sharp peak at the resonant frequency. For certain detunings of the harmonic wavelength from the resonance, this product results in a double-peak structure, where one peak is close to the harmonic frequency and the other is close to the resonance frequency.

This splitting is an evidence of the coherent emission owing to the following processes: the peak at the transition frequency is emitted due to the four-step process, where the wing of the harmonic line overlapping with the transition frequency is resonantly enhanced, and the peak at the exact harmonic frequency is emitted due to the three-step process, where the electrons from the continuum directly recombine to the ground state, as illustrated in Fig. 2. For brevity, we denote these peaks as RH and direct harmonic (DH), respectively.

Thus, by using a wavelength tunable driving laser and taking advantage of the wavelength locked RH emission (discussed above), we demonstrate that the DH could be separated from the RH by carefully detuning the driving laser wavelength from the resonant wavelength.

### Harmonic emission from the dressed-AIS

**Figure 3:**
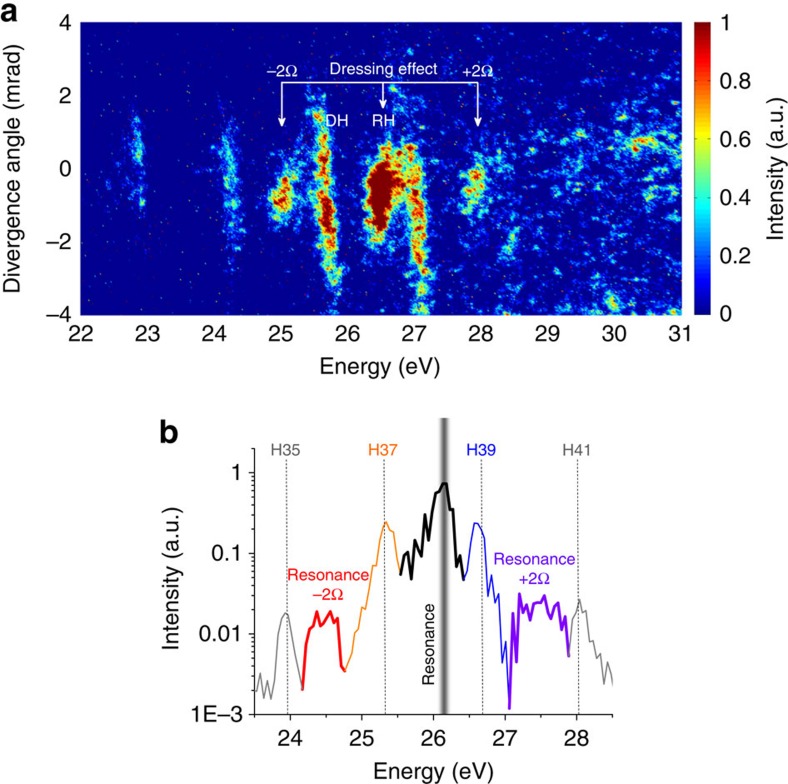
Separation of resonant harmonic and direct harmonic at detuned driving laser. (**a**) High-order harmonic spectrum of Sn^+^ using a driving laser with 1.785 μm central wavelength. This spectrum demonstrates that the resonant harmonic (RH) and the direct harmonic (DH) can be separated by detuning the driving laser wavelength. In this spectrum, two additional coherent harmonics are observed at ±2Ω around the RH, which are generated from the dressed autoionizing state (AIS). This spectrum is recorded at a driving laser intensity of ∼1.4 × 10^14^ W cm^−2^. (**b**) The harmonic spectrum calculated via 3D time-dependent Schrödinger equation for a model Sn^+^ ion in the laser field, showing good agreement with experimental observations.

Figure 3a shows the spectrum in the vicinity of the transition frequency for 1.785 μm driving wavelength. When the central driving laser wavelength is tuned from 1.75 to 1.785 μm, the RH and the DH are separated from each other. Moreover, with this off-resonance detuning, we reveal a never-considered feature of high-order harmonics. Namely, we demonstrate in Fig. 3a that when the driving laser wavelength is detuned, two additional harmonics appear at energies ∼25.0 eV and ∼27.9 eV. These satellite harmonics are located exactly at ±2Ω around the RH, where Ω is the photon energy of the driving laser. One can also notice that the divergence of both satellite harmonics is close to the divergence of the RH. We explain these observations as due to the generation of harmonics from the dressed AIS.

### Theoretical calculations

We perform simulations to model our experimental observations. Our theoretical study is based on the numerical solution of the 3D time-dependent Schrödinger equation for a model Sn^+^ ion in an external laser field. The details of the theoretical approach are described in the Supplementary Information. Figure 3b shows the calculated XUV spectrum near the resonance of tin. In this spectrum, we see the usual 37th (Orange line) and the 39th (Blue line) harmonics, and additional peaks in between, in agreement with the experimental results. The position of the additional peak at the red-side of the 39th harmonic (Black line) is close to the exact resonance. This splitting of the harmonic line near the resonance can be well-understood within the analytical theory^22^ as was described above. We also see that there are additional peaks (satellites) on the red side of the 37th (red line) and the 41st (violet line) harmonics. Gabor analysis is used to understand the origin of these peaks. The complete details of this analysis are given in the Supplementary Information. In short, the calculations indicate that opposite to the usual harmonics these satellites are emitted only when the central RH is emitted, thus when the AIS is populated and when the laser field is on. The AIS dressing naturally takes place only within the laser pulse, so these results indicate that the satellites emission involves the resonance with the dressed AIS. Thus, the calculations confirm that the satellite harmonics experimentally observed in Fig. 3a at ±2Ω around the RH are generated from the dressed-states of the autoionizing resonance.

### RH generation process

**Figure 4:**
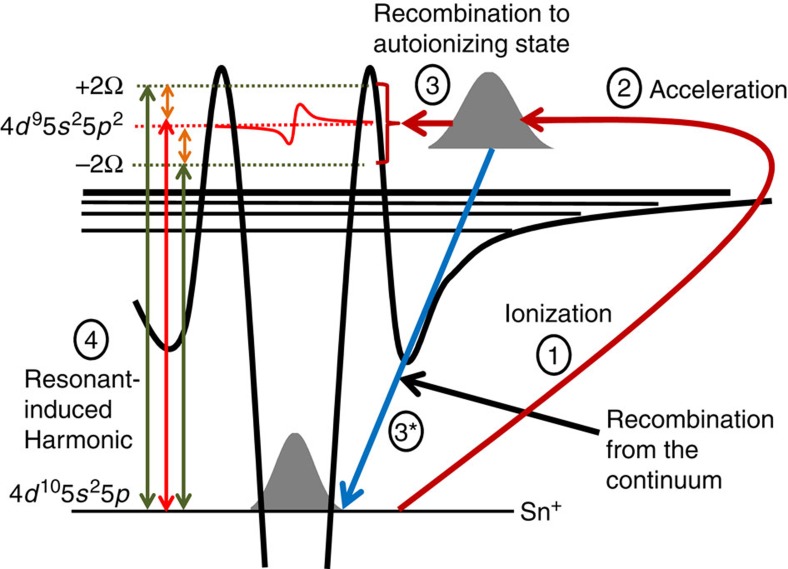
Schematic illustrations of electron quantum paths. Schematic diagram describing the four-step model of HHG from dressed AIS of Sn^+^. When high-intensity laser pulses (∼10^14^ W cm^−2^) interact with Sn^+^, an electron is detached from the ground state (Step 1). This electron is then accelerated in the continuum by the laser field (Step 2). In the third step, the continuum electron has two possible paths to follow. It can either recombine directly to the ground state (Step 3*) and generate DH, or it can be trapped so that the system finds itself in the AIS or dressed AIS (Step 3) and then relaxes to the ground state and emits the RH (Step 4). In the latter scenario, three coherent RHs are generated.

Previous studies of RH generation were focused at a single state (the AIS for the case of Sn^+^), which is in the multiphoton resonance with the driving laser^11^,^20^. However, our experimental observations show that RH generation can involve three different states: the actual AIS and the two dressed states located at ±2Ω around the resonant AIS. At the third step of the four-step model the system finds itself in one of these states and then, at the fourth step, it recombines to the ground state and emits one of the three RHs. This complete mechanism of RH generation is illustrated in Fig. 4. It is obvious that when the driving wavelength corresponds to the exact multi-photon resonance, the harmonics from these dressed states overlap with the neighbouring non-RH, and thus the two are difficult to differentiate (Fig. 2). However, these satellite harmonics can be distinguished from each other when the driving wavelength is detuned. Thus, this observation shows that the driving wavelength detuning from the resonance allows to observe the very pronounced separation of RH and DH, as well as the spectral peaks generated due to the dressed AIS.

Note that very pronounced enhancement of the harmonics neighbour to the resonant one was found theoretically in ref. 19. This theory considers a coherent superposition of ground and excited states formed by pumping by the RH, and thus assumes quite high intensity of this harmonic. The moderate intensity of RH in our experiments (in general, comparable to the one of the DH) prevents the direct application of this theory for their interpretation. However, our explanation of the process, presented in Fig. 4, can be understood as well in the terms of theories^18^,^19^, involving coherent superposition of states: the coherent superposition of the ground state and AIS appears as a result of the rescattering (as it was suggested in ref. 17), and then interacts with the laser field, demonstrating features predicted in ref. 19.

Our observations provide the accurate information of the quantum path that the electron follows in the vicinity of the AIS for RH generation. We have shown that for RH generation the electron quantum path is perturbed not only from the AIS but also from the dressed states, resulting in three coherent harmonic generation. In high-order harmonic attosecond science, pump-probe absorption spectroscopy has been recently started to study the light-matter interaction with dressed AIS^23^,^24^. However, the propagation and emission dynamics of the electron from dressed AIS have been highly ignored. Our observation of satellite harmonics from dressed AIS shows that the AIS dressing by the laser field should be taken into account to describe also the emission processes during the light-matter interaction for elements containing AIS. As AIS exist in different materials^25^, the present work will be applicable in general to study the influence of dressed states on electron quantum path in all these materials. As dressed states respond within the driving laser pulse, the nonlinear properties of the dressed AIS will provide the opportunity to study and control electronic motion at fast timescales, which will be an advantage as electrons remain in the AIS for only a few 100 attoseconds to a few femtoseconds.

## Methods

### Experimental set-up

The schematic diagram of the experimental setup used for HHG from laser-ablated plumes is shown in the Supplementary Fig. 1. The complete details of the mid-infrared laser source that was used for HHG can be found in ref. 26. In short, an uncompressed Ti:sapphire laser beam with a repetition rate 100 Hz, pulse duration of ∼210 ps and a central wavelength of ∼0.8 μm is divided into two beams. The first beam, called the prepulse (PP), is focused on to a solid tin target to generate low-density plume, which will be the nonlinear medium for harmonic generation. The PP intensity is maintained at ∼10^10^ W cm^−2^ over a circular spot of ∼200 μm in diameter. This intensity is used to ablate a plume from the target surface, placed inside a vacuum chamber having pressure ∼10^−5^ Torr. The second beam, called the main pulse (MP), is compressed to ∼42 fs and then seeded to an optical parametric amplifier (OPA; HE-TOPAS) for nonlinear frequency conversion. The output of this optical parametric amplifier, with energy∼750 μJ and pulse duration ∼51 fs, is then subsequently amplified with an amplification stage. This laser system is capable of producing 10 mJ of energy at 1.8 μm wavelength. The wavelength of these pulses are tuned close to the multiphoton resonance and then used for HHG, after a certain delay of 45 ns from the plume ablation. These high-order harmonics are then sent to a soft X-ray spectrometer, consisting of a flat-field Hitachi grating (1,200 lines per mm) and a multi-channel plate (MCP) followed by a phosphor screen. Finally, the harmonic spectrum detected by the MCP is then captured by a 16-bit camera (CMOS, PCO-edge).

### Coherence

When AIS are involved in RH generation, the common question that arises is whether RH emission is coherent or it is a spontaneous emission from the AIS. The coherent emission of RH can be checked simply by looking into the divergence of the harmonics on the MCP (Supplementary Fig. 2). The high-order harmonics have well-collimated emission that propagates collinearly with the driving laser field. However, spontaneous atomic or ionic transitions are emitted in 4π direction, and thus cover the complete width of the slit (1 cm) of the XUV spectrometer. In reality, this emission angle is limited by the size of the slit, which is ∼11 mrad. Therefore, harmonic emission can easily be differentiated from the spontaneous emission, as can be seen in Supplementary Fig. 2. Further, the divergence of the RH is observed to be much smaller than the divergence of the non-RH, generated with the three-step process (Fig. 3a). This is because the RH of Sn^+^ has well collimated emission owing to better phase matching than the harmonics generated with the three step process. The low divergence of the RH indicates that RH propagates with the non-RH in the same direction. The coherent RH emission occurs because of the short lifetime of the AIS involved in RH generation. The lifetime of the resonant state can be estimated with the line width of the AIS. The line width of the 4*d*^9^5*s*^2^5*p*^2^ (1D) ^2^D_5/2_ state is *Γ*=160 meV, which corresponds to the life time of ∼4.1 fs (*τ=ħ/Γ*). The similarity in divergence observed for the satellite harmonics further confirms the coherent emission of the RHs. Further information of the coherent RH emission can be found in ref. 8.

### Data availability

The data sets generated during and/or analysed during the current study are available from the corresponding author on reasonable request.

## Additional information

**How to cite this article:** Fareed, M. A. *et al*. High-order harmonic generation from the dressed autoionizing states *Nat. Commun.* **8**, 16061 doi: 10.1038/ncomms16061 (2017).

**Publisher’s note:** Springer Nature remains neutral with regard to jurisdictional claims in published maps and institutional affiliations.

## Supplementary Material

Supplementary Information
