# Supplementary material for: High-order harmonic generation from the dressed autoionizing states
Source: Nat Commun. 2017 Jul 17;8:16061. doi: 10.1038/ncomms16061 (PMC5520015; doi:10.1038/ncomms16061)
Supplement: Supplementary Information [file ncomms16061-s1.pdf]

File Name: Supplementary Information
Description: Supplementary Figures, Supplementary Methods and Supplementary References.

Supplementary Information

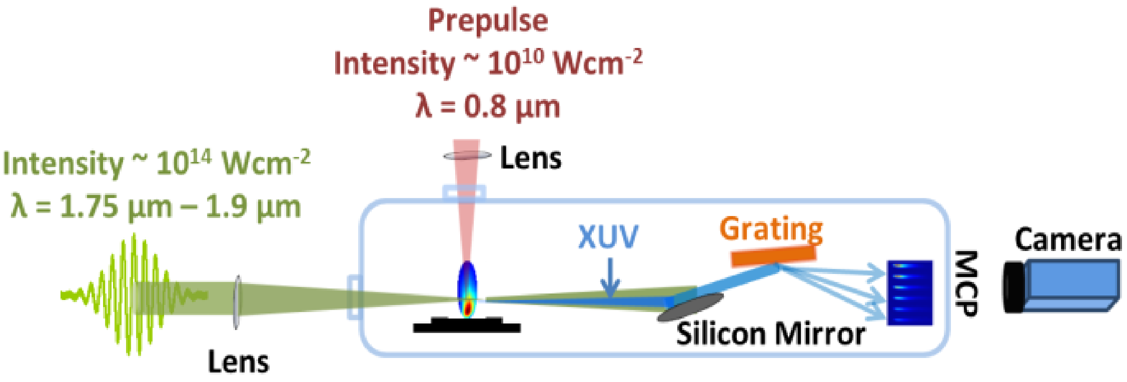

Supplementary Figure 1 | Experimental setup. Schematic diagram of the experimental setup used for high-order harmonic generation from laser-ablated plumes.

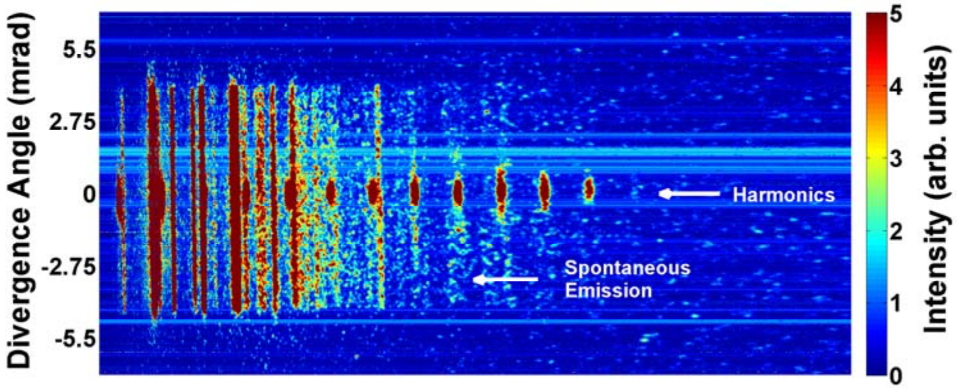

Supplementary Figure 2 | Comparison of divergence between coherent and non-coherent emission. Random spectra showing collimated harmonic emission and the spontaneous atomic or ionic emission from plasma, on the multi-channel plate.

Supplementary Methods:

In our calculations, the spectrum is found via numerical solution of the 3D time-dependent Schrödinger equation (TDSE) for the model system in the laser field. The numerical approach is presented in Ref. 1 and the model Sn^{+} potential is described in Ref. 2. Only short quantum path contribution to HHG is presented, and the long quantum path contribution is suppressed with the method suggested in Ref. 3. The peak laser intensity is $2\text{x}10^{14}$ Wcm^{-2} and the driving laser wavelength is 1.81 μm. We are using specific softly-truncated Gaussian temporal envelope of the laser pulse, namely, the field is:

$$E(t) = E_0 \exp\left[-\ln\left(\sqrt{2}\right)\left(\frac{t}{\tau}\right)^2 \right] f(t) \sin(\Omega t) \qquad \textbf{(1)}$$

Where $f(t)$ rises during time interval τ_{front}, then it is constant during time interval τ_{top}, and finally it falls during time interval τ_{front}:

$$f(t) = \begin{cases} \sin^2\left(\dfrac{\pi\left(t + \tau_{\text{front}} + \tau_{\text{top}}/2\right)}{2\tau_{\text{front}}}\right), & -\tau_{\text{front}} - \tau_{\text{top}}/2 < t \le -\tau_{\text{top}}/2 \\ 1, & -\tau_{\text{top}}/2 < t \le \tau_{\text{top}}/2 \\ \sin^2\left(\dfrac{\pi\left(t - \tau_{\text{front}} - \tau_{\text{top}}/2\right)}{2\tau_{\text{front}}}\right), & \tau_{\text{top}}/2 < t \le \tau_{\text{front}} + \tau_{\text{top}}/2 \\ 0, & \text{otherwise} \end{cases} \qquad \textbf{(2)}$$

In our calculations, the FWHM of the Gaussian (2τ) is 18 fs, τ_{front} is 3 optical cycles (18.1 fs) and τ_{top} is 8 optical cycles (48.3 fs). So the pulse has almost Gaussian envelope. The envelope is softly truncated by the function *f(t)* for $t > \tau_{\text{top}}/2$. However, this truncation takes place only far at the wings of the Gaussian because under the used parameters $\tau_{\text{top}}/2 \approx 2.6\tau$ so the pulse intensity is softly truncated when it is less than 1% from its maximum.

Thus, the used field has long temporal wings, in contrast to $\sin^2$ envelope widely used in numerical calculations. In this sense our field at least qualitatively reproduces the experimentally used one. We will show below that it is very important to reproduce the presence of the laser pulse temporal wings in the calculations of the dressed AIS states. The truncation of the Gaussian is necessary to have the field exactly equal to zero at the start of the numerical TDSE solution. Moreover, the used field satisfies the $\int_{-\infty}^{+\infty} E(t)dt = 0$ condition.

The calculated spectrum is shown in the main article in **Fig. 3 (b)**.

To study the temporal dynamics of the XUV emission, we use Gabor analysis. Namely, using the TDSE solution we find the microscopic dipole *d(t)* and calculate its spectrum:

$$f(\omega) = \int_{-\infty}^{+\infty} d(t)\exp(i\omega t)dt$$

Then, we find the inverse Fourier transform of this spectrum within the spectral range from ω_1 to ω_2 (the Gabor transform):

$$\tilde{d}_{\omega_1,\omega_2}(t) = \frac{1}{2\pi}\int_{\omega_1}^{\omega_2} f(\omega)\exp(-i\omega t)d\omega$$

In **Supplementary Fig. 3 (a & b)** we present the intensity of this signal $\tilde{I}_{\omega_1,\omega_2}(t) = \left|\tilde{d}_{\omega_1,\omega_2}(t)\right|^2$ for several spectral ranges $[\omega_1, \omega_2]$.

As we have shown in the main article in **Fig. 3 (b)**, additional peaks appear in the spectrum at the resonant frequency and at this frequency $\pm 2\Omega$. To understand their origin, we make the Gabor analyze, namely we select several peaks from the spectrum and study when they are emitted. In **Supplementary Fig. 3 (a)**, we see that harmonics H37 and H39 are emitted mainly within the laser pulse as it should be for the non-resonant harmonic. The resonant emission (black line in **Supplementary Fig. 3b**) takes place at the falling edge of the pulse and after the pulse. This behavior is typical for the resonant XUV[4], even the exponential decrease of the emission caused by the decay of the autoionizing state (AIS) can be seen after the end of the laser pulse. The satellites at the resonant frequency $\pm 2\Omega$ (red and violet line in **Supplementary Fig. 3b**) are emitted only during the falling edge of the laser pulse, but not after the pulse.

Taking into account this behavior, one can explain the emission of the satellites at the resonant frequency $\pm 2\Omega$ by the resonance with the dressed AIS. Indeed, the AIS is mainly populated at the falling edge and after the pulse (see usual resonant emission, black line in **Supplementary Fig. 3 b**) but the dressed states exist only inside the laser pulse, not after it. Thus the emission due to the resonance with the dressed AIS should be temporally confined within the falling edge of the pulse but not after it.

The correct reproduction of the edges of the laser pulse is thus very important for the calculation of the emission from the dressed AIS. Using the $\sin^2$ temporal envelope in the calculations, we found far less pronounced emission from the dressed AIS, because the temporal interval where the AIS is populated and the laser field dresses it is much shorter. Our softly-truncated Gaussian envelope is closer to the real laser pulse. However, the wings of the laser pulse can be more pronounced (of even some pedestal of the laser pulse can be important), so this explains the difference between the experimental and calculated spectra.

Note that alternatively the peaks at the resonant frequency ±2Ω can be attributed to the resonance between the (usual) AIS and the dressed ground state. However, the population of the ground and dressed ground state can be easily tracked in the TDSE numerical solution. These populations differ by more than 3 orders of magnitude, while the intensity of the usual resonant emission (black line in **Fig. 3 (b)** of the main text and "resonant emission involving dressed states" (red and violet lines)) are closer, namely they differ by approximately 2 orders. This allows us to conclude that the dressed ground state cannot explain the observed emission. Moreover, in general the AIS should be more affected by the laser than the ground state, so the dressed AIS is more populated than the dressed ground state.

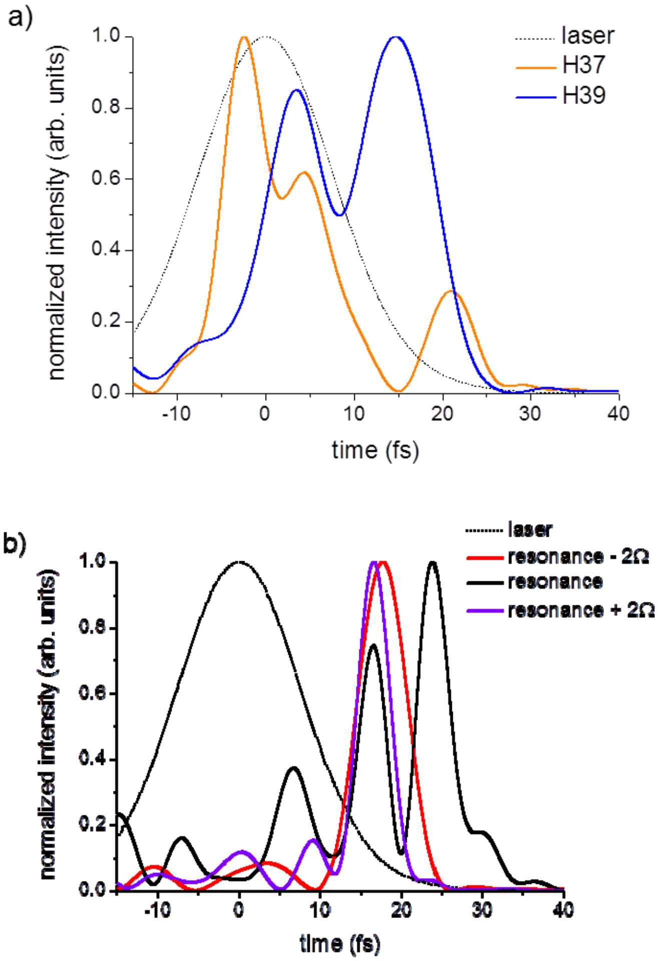

Supplementary Figure 3│Gabor transform of calculated harmonic spectrum. Gabor transforms of several spectral peaks shown in **Fig. 3(b)** in the main text; the transforms show when 37th and 39th harmonics **(a)** and XUV at the transition frequency and at this frequency ±2Ω are emitted **(b)**. Dotted black line shows the driving field envelope. Every curve is renormalized to its maximum. The spectral ranges used for the Gabor transforms are shown in **Fig. 3(b)**, in the main text.

Supplementary References

1. Strelkov, V. V, Sterjantov, A. F., Shubin, N. Y. & Platonenko, V. T. XUV generation with several-cycle laser pulse in barrier-suppression regime. *J. Phys. B* **39**, 577–589 (2006).
2. Ganeev, R. A. et al. Experimental and theoretical studies of two-color-pump resonance-induced enhancement of

odd and even harmonics from a tin plasma. *Phys. Rev. A* **85**, 023832 (2012).

3. Strelkov, V. V., Khokhlova, M. A., Gonoskov, A. A., Gonoskov, I. A. & Ryabikin, M. Y. High-order harmonic generation by atoms in an elliptically polarized laser field: Harmonic polarization properties and laser threshold ellipticity. *Phys. Rev. A* **86**, 013404 (2012).
4. Tudorovskaya, M. & Lein, M. High-order harmonic generation in the presence of a resonance. *Phys. Rev. A* **84**, 013430 (2011).
